# THE IMPACT OF DIABETES AND POOR ORAL HEALTH ON COGNITION: IMPLICATIONS TO IMPROVE DENTAL CARE ACCESS

**DOI:** 10.1093/geroni/igz038.2261

**Published:** 2019-11-08

**Authors:** Bei Wu, Huabin Luo

**Affiliations:** 1 New York University, New York, New York, United States; 2 University of Alabama at Birmingham, Greenville, North Carolina, United States

## Abstract

This study examined the joint effects of diabetes and poor oral health on cognitive function among older adults aged 60 years or older in the U.S. We analyzed data of 2,937 participants from the National Health and Nutrition Examination Survey (2011-2014). We investigated the interaction effects between diabetes and significant tooth loss, i.e. differences among the following four groups: 1=neither of the two conditions, 2=non-diabetic but with tooth loss, 3=diabetic but no tooth loss, and 4=both conditions. Significant interaction effects were found in our study. Having either diabetes or significant tooth loss was associated with lower cognitive function. When the two conditions were both present, the negative effects were much stronger than the total effects from either one of the two conditions. The additional loss of cognitive function resulting from multiple health conditions illustrates the importance of improving access to dental care for older adults in the U.S.

